# Transmission of bee-like vibrations in buzz-pollinated plants with different stamen architectures

**DOI:** 10.1038/s41598-021-93029-7

**Published:** 2021-06-29

**Authors:** Lucy Nevard, Avery L. Russell, Karl Foord, Mario Vallejo-Marín

**Affiliations:** 1 Biological & Environmental Sciences, University of Stirling, Stirling, FK9 4LA UK; 2 Department of Biology, Missouri State University, Springfield, MO 65897 USA; 3 Minnesota Extension, University of Minnesota, St Paul, MN 55108 USA

**Keywords:** Pollination, Biomechanics, Plant physiology

## Abstract

In buzz-pollinated plants, bees apply thoracic vibrations to the flower, causing pollen release from anthers, often through apical pores. Bees grasp one or more anthers with their mandibles, and vibrations are transmitted to this focal anther(s), adjacent anthers, and the whole flower. Pollen release depends on anther vibration, and thus it should be affected by vibration transmission through flowers with distinct morphologies, as found among buzz-pollinated taxa. We compare vibration transmission between focal and non-focal anthers in four species with contrasting stamen architectures: *Cyclamen persicum, Exacum affine, Solanum dulcamara* and *S. houstonii*. We used a mechanical transducer to apply bee-like vibrations to focal anthers, measuring the vibration frequency and displacement amplitude at focal and non-focal anther tips simultaneously using high-speed video analysis (6000 frames per second). In flowers in which anthers are tightly arranged (*C. persicum* and *S. dulcamara*), vibrations in focal and non-focal anthers are indistinguishable in both frequency and displacement amplitude. In contrast, flowers with loosely arranged anthers (*E. affine*) including those with differentiated stamens (heterantherous *S. houstonii*), show the same frequency but higher displacement amplitude in non-focal anthers compared to focal anthers. We suggest that stamen architecture modulates vibration transmission, potentially affecting pollen release and bee behaviour.

## Introduction

Insects use substrate-borne vibrations in a range of ecological contexts, including conspecific communication and the detection of prey and predators^1,2^. These vibrations are often produced and detected on plant material, and the physical properties of the plant substrate, such as stem stiffness or leaf thickness, often affect vibration propagation^3–5^. Approximately 6–8% of angiosperms are buzz-pollinated, relying on substrate-borne vibrations (floral buzzing), typically produced by bees, to release pollen from flowers with specialised morphologies^6,7^. While buzz pollination is a widespread plant–insect interaction common in agricultural and natural ecosystems, its biomechanical aspects remain understudied compared to other insect vibrations, such as those used in communication.

More than half of all bee species can buzz to collect pollen, and the behaviour is thought to have evolved approximately 45 times within bees (Anthophila)^8^. During buzz pollination, the bee typically clutches the anthers with its mandibles and produces thoracic vibrations using the indirect flight muscles^6,9^. These vibrations are transmitted to the flower, triggering pollen release. Most buzz-pollinated flowers have tubular anthers that dehisce only via small apical pores or slits, i.e., poricidal anthers, through which small, dry pollen grains are released during floral vibrations^10^. Moreover, some species with longitudinally dehiscent anthers have evolved floral morphologies which also rely on floral buzzing for pollen release. For example, the modified corolla of some *Pedicularis* species encloses the anthers in a tube, which thus functions analogously to an individual poricidal anther^11^. Furthermore, many species with non-poricidal anthers and apparently accessible pollen, e.g., *Rosa* or *Begonia* species, are often buzzed by bees, presumably maximizing pollen collection^12,13^. The interaction between flower and vibrating bee is thus very widespread, emphasising the importance of studying floral vibrations in detail across plant lineages.

Similar to the study of vibrations used for insect communication, the functional study of floral vibrations can be divided into three major stages: (1) the production of vibrations by the bee, (2) the propagation of these vibrations through the bee-flower coupled system, and (3) the effect of vibrations on pollen release^7,14^. Most work to date has focused on (1) bee buzzing behaviour and/or (3) pollen release. Bees produce floral vibrations which vary in duration, frequency (oscillations per unit time) and amplitude, the primary components with which vibrations can be described^7,15^. Vibration amplitude, whether measured as displacement, velocity, or acceleration, has a significant and positive effect on pollen release: higher amplitude vibrations release more pollen^16–18^. In contrast, the effect of vibration frequency on pollen release appears to be weaker within the natural range of bee buzzes ~ 100–400 Hz^17,19,20^, although vibrations at much higher frequencies than those produced by bees do result in the release of more pollen^10,21^.

Buzz-pollinated flowers are morphologically diverse, yet the intra-floral transmission of vibrations across a range of species has been rarely investigated. The structure of the androecium, e.g., the spatial arrangement of the anthers, is likely to affect transmission of vibrations. Here we follow Endress^22^ and define stamen architecture as the relative sizes of stamens, their degree of fusion, and their spatial and functional connections^23^. Many taxa with poricidal anthers have converged on a stamen architecture in which equally sized anthers are held tightly together forming an anther cone as in *Solanum dulcamara* L. and *S. lycopersicum* L.^24^*.* Interestingly, this anther cone morphology has evolved independently in many other groups of flowering plants^24–26^. During floral buzzing, bees often use their mandibles to hold only a subset of the anthers in the flower^27^. In species with tightly arranged anthers, bee vibrations applied to one or a few anthers are likely to be effectively transmitted to the rest of the anther cone. In contrast, in buzz-pollinated species with anthers presented more loosely (e.g., most *Melastomataceae*, *Solanum elaeagnifolium* Cav., *S. sisymbriifolium* Lam.), applying vibrations to a subset of focal anthers might limit transmission to non-focal anthers in the same flower.

This potential difference in vibration transmission between focal and non-focal anthers is perhaps best exemplified in heterantherous species, in which two or more morphologically distinct sets of anthers occur in the same flower^26^. In some heterantherous species, the two anther sets perform different functions, with long anthers contributing disproportionately to pollination (pollinating anthers) and short anthers (feeding anthers) being the focus of attention of buzz-pollinating bees^28–30^. A recent study has shown that pollinating and feeding anthers of heterantherous *Solanum* differ in natural frequency, also known as the first mode of vibration, which is the lowest frequency at which a material object vibrates when disturbed. This is likely a result of differences between anther types in biomechanical properties, including size and shape^31^. Despite the potential for differences in anther and floral characteristics, such as those described above, to affect the transmission of vibrations in buzz-pollinated flowers, few studies have explicitly compared floral vibrations across different floral morphologies.

Here, we used a mechanical transducer to apply bee-like artificial vibrations to focal anthers, simultaneously measuring the vibration frequency and displacement amplitude at the tips of focal and non-focal anthers of the same flower in two axes using high-speed video analysis (6000 frames per second). We used four buzz-pollinated species with contrasting floral and poricidal anther morphologies: *Cyclamen persicum* Mill. (Primulaceae)*, Exacum affine* Balf. ex Regel (Gentianaceae)*, Solanum dulcamara* and *S. houstonii* Dunal (Solanaceae). The arrangement of anthers within these flowers varies from a tight cone (*S. dulcamara*) to a loose, heterantherous assemblage (*S. houstonii*). We ask the following questions: (i) Does the dominant frequency of vibrations change between focal and non-focal anthers in these flowers? (ii) Does vibration amplitude (measured as displacement amplitude) change between focal and non-focal anthers? (iii) Do vibration characteristics depend on plant species and/or the characteristics of the applied vibration? Based on previous work suggesting conservation of frequency properties during buzz pollination^32,33^, but changes in amplitude as vibrations travel through the flower^34,35^, we predict that vibration amplitude, but not frequency, will be conserved from focal to non-focal anthers less faithfully in flowers with looser anther arrangements. High-speed video requires no physical interference with the system and is an alternative to other non-contact methods to study vibrations across complex structures such as laser scanners. Our study allows us to quantify and compare the transmission of floral vibrations in flowers with different types of stamen architectures and may be useful for future work on the function and evolution of different floral morphologies among buzz-pollinated plants.

## Materials and methods

### Study system and plant material

**Figure 1:**
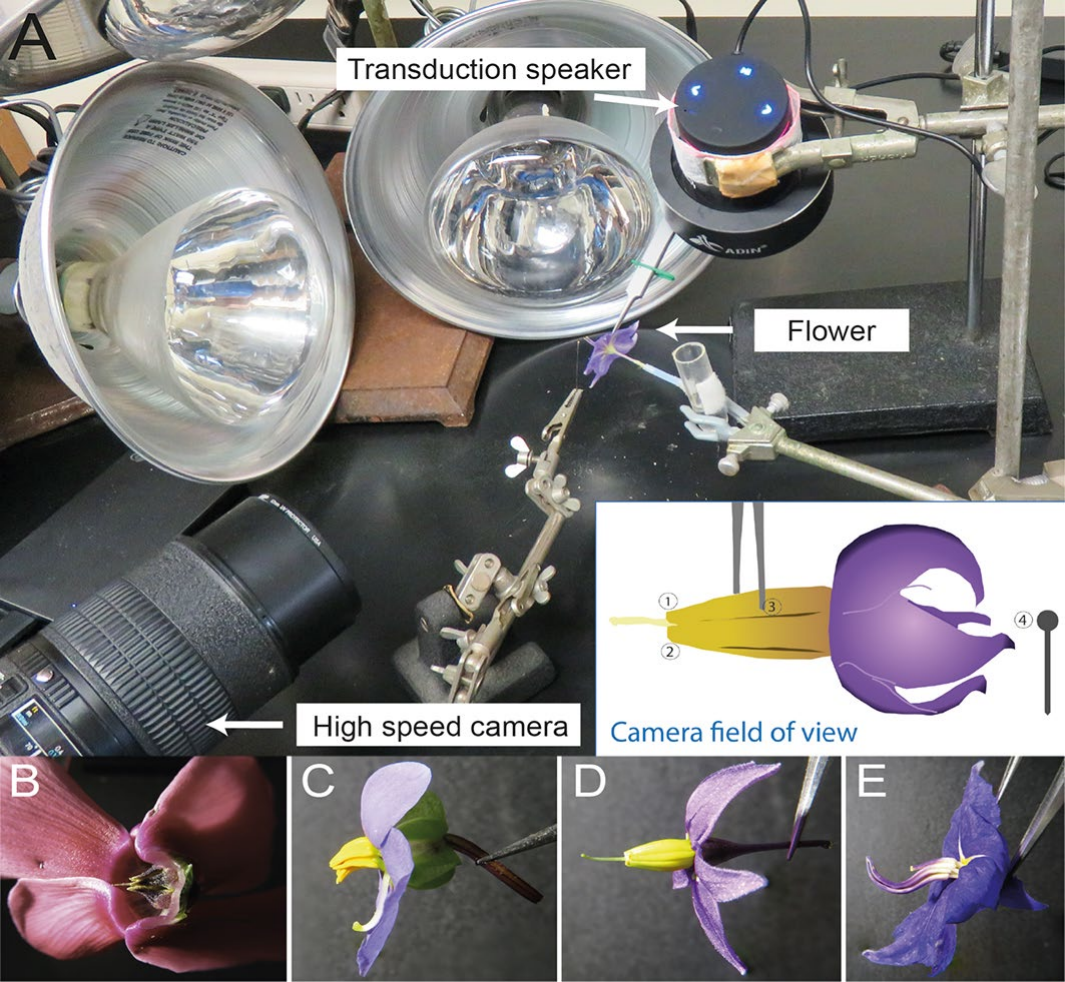
(**A**) Experimental set up of the artificial vibration playback system. The inset shows a diagram of the camera field of view. 1: Focal anther tip; 2: non-focal anther tip; 3: forceps tip; 4: insect pin for calibration*.* Lateral view of the flowers of the four species studied here: (**B**) *Cyclamen persicum*. (**C**) *Exacum affine*. (**D**) *Solanum dulcamara*. (**E**) *Solanum houstonii*.

We studied flowers of four species from three families: *Cyclamen persicum* Mill. (Primulaceae)*, Exacum affine* Balf.f. ex Regel (Gentianaceae)*, Solanum dulcamara* L. and *S. houstonii* Martyn (Solanaceae). These species have contrasting stamen architectures and are nectarless, offering only pollen as a reward to floral visitors (Fig. 1B–E). *Cyclamen persicum* flowers have poricidal anthers fused by the filaments (connate) into a symmetrical conical shape^36^. Together with other *Cyclamen* species, *C. persicum* was historically presumed to be buzz-pollinated, based on the presence of poricidal anthers. However, buzz-pollinating visitors are rarely observed in wild populations and its main pollinators are often moths, hoverflies and small bees^36^. *Exacum affine* has unfused (distinct), slightly curved, poricidal anthers and is primarily buzz-pollinated^37,38^. *Solanum dulcamara* has poricidal anthers which are fused into a single cone (connivent), with very short filaments^24^. *Solanum houstonii* has unfused (distinct), curved, poricidal anthers and is heterantherous: it has two short anthers (feeding anthers) presumed to be mainly involved in attracting and rewarding pollinators, and three longer, S-shaped anthers (pollinating anthers) presumed to contribute disproportionally to pollination^27^. Both *Solanum* species are visited and buzz-pollinated by bees of diverse sizes and morphologies, although the most effective pollination of *S. houstonii* is presumably performed by relatively large bees, such as *Bombus sp., Centris sp.* and *Xylocopa* sp.^24,27,39–41^, (L. N. pers. obs.).

Plants were purchased as full-grown plants or grown from seeds or cuttings in university greenhouses in Tucson, AZ and Pittsburgh, PA. *Cyclamen persicum* plants were sourced from Lowe’s Home Improvement. *Exacum affine* plants of three varieties (Champion Blue, Royal Blue, Little Champ Blue) were sourced from the wholesaler Fred C. Gloeckner & Co. *Solanum dulcamara* cuttings were collected from wild populations in Pittsburgh, PA. *Solanum houstonii* seeds were sourced from the Sonoran Desert Museum, Tucson, AZ; originally collected from wild populations in Mexico.

### Synthesis and playback of vibration signals

The experiment consisted of generating synthetic vibrations and applying them to individual flowers using a vibration transducer mechanical system, to analyse the vibrational properties of different parts of the flower. Flowers used in experiments were as fresh as possible, usually newly opened on the plant each morning of the experiment. Synthetic vibrations consisted of 1 s pure tone signals of fixed amplitude with a 10 ms fade in and 10 ms fade out. We conducted two sets of experiments. In the first set, we varied relative amplitude of the signal, while keeping frequency constant. Signals were generated by creating a sine wave with frequency of 350 Hz, using the Tone function in Audacity ver. 2.1.0 (http://audacityteam.org/) and saved as a single-channel audio file (WAV) at 44.1 kHz sampling rate. We used four relative amplitude levels: (in dB): − 15, − 10, − 5 and 0. The absolute displacement amplitude of the vibrations applied to the flower in each of these treatments was calculated using the observed displacement of the forceps tips (see “Digitising video files and time series analysis” section). For each amplitude, we conducted 2–3 playback replicates per species in each of two species selected based on flower availability (*Exacum affine* and *Solanum houstonii*). Overall, in this experiment, we used 18 flowers, 9 of each species. In the second experiment we generated signals as above with constant relative amplitude (0 dB) but with different individual frequencies (150, 200, 250, 300, 350, 400, 450, and 500 Hz). The frequency values we used reflect the range of frequencies recorded from bees vibrating on buzz-pollinated flowers^19,20,42^. For each frequency, we conducted 3–4 playback replicates for each of the four species studied, depending on flower availability. Overall, in this experiment, we used 104 flowers, 26 of each of the four species.

We played back each vibration signal using a Zoom H2 audio recorder (Zoom Corporation; Tokyo, Japan) connected to a vibration speaker (Adin S8BT 26 W). The output volume of the Zoom H2 and vibration speaker was kept constant, except as noted in the “Results” section. The vibration speaker was modified as described in Brito et al.^43^ to transduce the vibrations to the flower by fixing a metal rod to the vibrating plate of the speaker and attaching a pair of very fine tipped forceps (Fine Science Tools, Dumont #5 Biology Tip Inox Forceps) to the end of the rod. The forceps were used to hold 1–2 anthers (the short anthers in the case of *Solanum houstonii*) (see Fig. 1A for setup). Individual flowers were placed in floral water tubes, with the stamen’s long axis parallel to the ground, i.e., flowers were kept horizontal to the ground as they would be perceived by a pollinator directly approaching the centre of the flower. The movement of the forceps was thus perpendicular to the anthers. The forceps were clamped at approximately the same position (1/4 of the anther length from the connection with the filament) on the anthers for each trial. In trials on *C. persicum*, one petal was cut away to allow visualisation of the anthers. A fresh flower was used for each replicate such that each flower was vibrated only once and we collected data sequentially for each amplitude level or frequency before moving to the next set of replicates to control for effects of time of day on vibration characteristics.

### High speed digital imaging

To analyse the vibration of different parts of the flower simultaneously, we used high-speed digital imaging, which allowed us to simultaneously track the movement of captured objects along two dimensions at different locations of the image frame. We recorded the vibrating flowers at 6000 frames per second (fps; 1280 × 512 pixels) against a black background using a FASTCAM SA-8 camera (Photron, San Diego, California USA) and halogen bulbs for illumination. Recording started before the vibration playback began and captured the whole 1 s vibration. An entomological pin (size 1) was kept in shot for videos, to enable size calibration and consistency in video tracking output across different videos (Fig. 1).

### Digitising video files and time-series analysis

All video footage was analysed in two dimensions using the *DLTdv7* digitising tool^44^ in MATLAB 9.6 (R2019a; MathWorks Inc). Recordings were 730 ms long on average. This digitising tool allows point tracking in high-speed video footage^45^, and we used it to generate a time series of *x–y* coordinates for each tracked point. For each video, we simultaneously tracked three points through time to extract vibrational information measured as displacement: (1) The tip of the forceps, hereafter *control*. This allowed us to empirically obtain frequency and displacement amplitude of the input vibrations transduced to the flowers, and to account for variation in volume playback introduced during the experiment. (2) The tip of the anther held by the forceps, hereafter the *focal anther.* (3) The tip of the anther furthest away from the focal anther, hereafter the *non-focal anther*. In a few cases, it was not possible to track all three points for each sample due to obstruction of the control point by other parts of the flower or due to low light. All three points were reliably tracked in 87 out of 122 samples.

The x–y time series data was analysed using the *seewave* package^46^ in R ver. 4.0.2 (R Core Development Team, 2020). Displacement values (calibrated to mm, using the insect pin described previously as a reference for size) were calculated for x- and y-axes, by zero-centring the data. These x–y displacements were used to obtain an overall measure of displacement magnitude defined as (x-displacement^2^ + y-displacement^2^)^1/2^. We used a high pass filter of 80 Hz using the *fir* function (Hanning window, window length = 512 samples). For each digitised recording, a section of 100 ms in the middle of each time series was selected, where the vibration was more stable (approximately from 0.3 to 0.4 s for every sample). Twelve digitised samples which were too short were removed from the dataset, leaving 75 samples remaining as the final total sample size. From these 100 ms sections (sampled at 44,100 samples per second), we computed the frequency spectra using the function *spec* (using power spectral density) and calculated the dominant frequency using the function *fpeaks* (nmax = 1). We also estimated peak displacement amplitude (D_P_), peak-to-peak displacement (D_P-P_), and Root Mean Squared displacement (D_RMS_) using the functions *max* (on absolute values), *max – min*, and *rms*, respectively. These are commonly used parameters describing vibration properties in buzz pollination (Vallejo-Marín 2019). For example, the dominant frequency is the frequency of the sinusoidal component with the highest relative amplitude, while D_RMS_ reflects the overall energy content of a vibration (Sueur 2018).

### Statistical analysis

We evaluated the correlation between the different measurements of displacement amplitude (D_P_, D_P-P_, D_RMS_), and between displacement in the x-, y-axis and v-vector using Pearson moment correlations. We assessed the association between the characteristics of the input vibrations applied by the forceps (dominant frequency and D_RMS_) and those measured at the anther tips using linear models fitted with the function *lm*. In these models, vibration dominant frequency or D_RMS_ were used as the response variable, and input vibration (at the forceps tip), anther type (non-focal and focal anthers) and species as the explanatory variables. For each model, diagnostics were produced using the package DHARMa^47^. For those which showed significant outliers, models were re-created without these data points. The statistical significance of effects remained similar and therefore we kept the full data set for the final analysis. Statistical significance of the main effects and their interactions were assessed using Type III sums of squares using the package *car*^48^. Model predictions were plotted using *plot_model* (type = pred) in the package *sjPlot*^49^. All statistical analysis was performed in R 4.0.2 (R Core Development Team 2020).

### Ethics

All plant collection and material used in this study adheres to current state and federal US legislation.

## Results

### Frequency of anther vibrations

**Figure 2:**
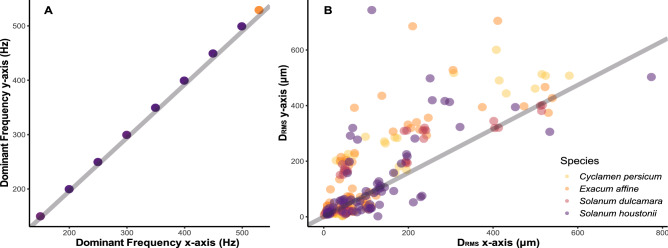
Measurement of (**A**) frequency (dominant frequency, Hz) and (**B**) root mean square amplitude (D_RMS_, μm) in either the x- or y-axis. Grey lines indicate 1:1 relationship.

**Figure 3:**
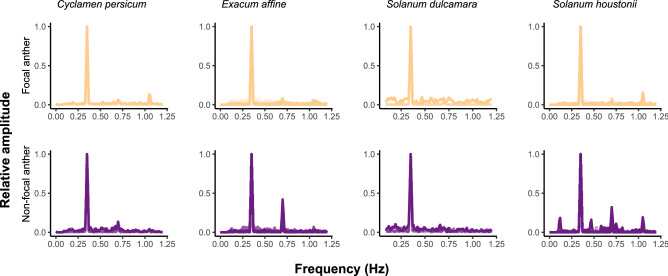
Frequency spectra for focal (top row) and non-focal anthers (bottom row) of four plant species in response to artificial vibrations applied in the focal anther using the vibration playback system shown in Fig. 1. The frequency of the input vibration was 350 Hz.

The dominant frequencies measured in the x- and y-axis were highly correlated across all samples. (Pearson’s correlation *r:* 0.98, df: 266, p < 0.001) (Fig. 2A). Dominant frequency across anthers and plant species ranged from 150 to 529 Hz (Fig. S3). Forceps dominant frequency was the only significant predictor of anther dominant frequency in our linear model (p < 0.001, Table 1A) and we found no effect of either anther type or plant species (i.e., anther arrangement) on the dominant frequency of vibrations measured at the tips of anthers (p > 0.05, Table 1A). In other words, the dominant frequency did not change as vibrations were transmitted through the flowers from the forceps. The overall frequency spectra were also similar between species and anther types, with very few harmonics in any of the vibrations (Fig. 3).

**Table 1:** Parameter estimates of the linear models fitted with either dominant frequency (Hz) or D_RMS_ as response, and forceps dominant frequency or D_RMS_, anther type, and species as explanatory variables.

	Estimate	Std. error	*p*-value*	*p*-value**
**A. Dominant frequency (Hz)**				
Forceps dominant frequency	1.000e+00	1.552e−16	** < 0.001**	
Anther (non-focal)	3.459e−14	3.077e−14	0.263	
Species				
(*Exacum affine*)	6.634e−14	4.279e−14	0.123	
(*Solanum dulcamara*)	7.741e−14	4.711e−14	0.103	
(*Solanum houstonii*)	6.576e−14	4.446e−14	0.141	
**B. Displacement amplitude D**_**RMS**_** (µm)**				
Forceps D_RMS_	1.012	0.045	** < 0.001**	** < 0.001**
Anther (Non-focal)	− 16.825	9.464	0.078	0.078
Species				0.13
(*Exacum affine*)	− 2.68	7.917	0.735	
(*Solanum dulcamara*)	− 2.969	8.775	0.736	
(*Solanum houstonii*)	12.441	8.05	0.124	
Forceps D_RMS_: Anther (non-focal)	0.202	0.064	**0.002**	**0.002**
Anther (non-focal): Species				** < 0.001**
Anther: (*Exacum affine*)	44.754	11.306	** < 0.001**	
Anther: (*Solanum dulcamara*)	1.31	12.303	0.915	
Anther: (*Solanum houstonii*)	40.533	11.792	** < 0.001**	

*P-value of explanatory variable in linear model.

**P-value calculated using Type III sums of squares. Sample size is 150 for both models.

### Amplitude of anther vibrations

All three measures of displacement amplitude differed slightly between the x- and y-axis across all samples, including in the forceps (Table 2). The average amplitude was higher in the y-axis, particularly in D_P_ and D_P-P_ (Table 2). Axes were nonetheless strongly correlated for all measures of amplitude: D_P_ (r: 0.81, df: 266, p < 0.001); D_P-P_ (r: 0.82, df: 266, p < 0.001); D_RMS_ (r: 0.79, df: 266, p < 0.001) (Fig. 2B for D_RMS_ correlations). Therefore, we used the vector magnitude (see “Materials and methods” section for details) for downstream analysis on amplitude, to capture variation in displacement in both x and y axes.

**Table 2:** Summary statistics across all samples of three measures of displacement amplitude (µm) of both focal and non-focal anthers combined.

Axis of measurement	X-axis		Y-axis		Vector	
Amplitude (µm)	Range	Mean ± s.e	Range	Mean ± s.e	Range	Mean ± s.e
D_P_	4.04–1500	199 ± 13.6	3.39–1340	230 ± 15.4	16.4–1030	174 ± 10.3
D_P-P_	7.37–2400.7	376 ± 25.1	6.21–2570	437 ± 29.8	32.7–1840	316 ± 18.8
D_RMS_	1.17–774	114 ± 8.21	1.77–744	130 ± 9.54	5.35–363	70.3 ± 4.36

The axis of measurement indicates whether the displacement was measured in the x-axis, the y-axes, or the resulting vector calculated from the combined x–y displacement (see “Materials and methods” section).

*D*_*P*_ peak displacement amplitude, *D*_*P-P*_ peak-to-peak displacement amplitude, *D*_*RMS*_ root mean square displacement amplitude.

We extracted three measures of displacement amplitude: D_P,_ D_P-P,_ and D_RMS_. D_P_ across anther types and species ranged from 16.4 µm to 1030 µm (mean 195), D_P-P_ ranges from 39.3 to 1840 µm (mean 353), D_RMS_ ranged from 6.94 to 363 µm (mean 77.8). The highest displacements for all measures were from vibrations in the non-focal anther of *S. houstonii* (heterantherous and loosely arranged stamens), and the lowest were from the non-focal anther of *C. persicum* (stamens fused in a cone). All three measures of displacements were strongly correlated across all trials: D_P_ and D_P-P_ (r: 1, df: 179, p < 0.001); D_P_ and D_RMS_ (r: 0.98, df: 179, p < 0.001); D_P-P_ and D_RMS_ (r: 0.99, df: 179, p < 0.001). D_RMS_ was used for all further amplitude analysis.

**Figure 4:**
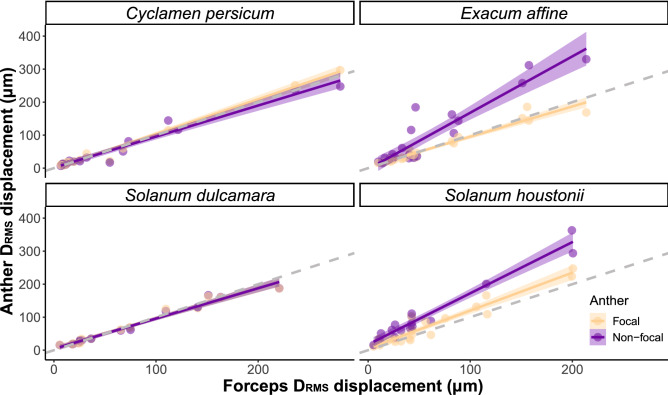
Linear model estimates and data points for displacement (vector D_RMS_, µm) of focal (yellow symbols) and non-focal anther (purple symbols), against forceps displacement (vector D_RMS_, µm) in four plant species. Values for the vector are calculated from the x- and y-axes (see “Materials and methods” section for details). Grey dashed line indicates a linear relationship with slope = 1.

We found a significant interaction between anther type and input D_RMS_ (measured at the forceps) on anther displacement (vector D_RMS_), with displacement in non-focal anthers generally increasing more rapidly with input amplitude than in focal anthers (Table 1B, Fig. 4). We also found a significant interaction effect between anther type and plant species on the displacement amplitude (vector D_RMS_) of vibrations (p < 0.001, Table 1B, Fig. 4), with higher displacements in the non-focal anthers of *E. affine* (coefficient = 42.87) and *S. houstonii* (coefficient = 46.11) (both species have loosely arranged stamens), compared to focal anthers of *C. persicum*, which has a fused stamen cone (Table 1B, Fig. 4). Separate analyses of the x- and y-axes both showed significant interactions between anther type and plant species (p < 0.005) (Figs. S5, S6, Tables S1, S2). When we calculated the disparity in D_RMS_ (vector) between the forceps and the anther, the mean difference across both anther types in *C. persicum* and *S. dulcamara* (both with fused stamen cones) was close to zero (Table 3). In contrast, the mean differences (disparity in D_RMS_ between the forceps and anther) for the non-focal anthers of *E. affine* and *S. houstonii* were 36.6 µm and 55.1 µm respectively, and for the focal anther of *S. houstonii* it was 13.8 µm (Table 3).

**Table 3:** Difference in displacement amplitude in µm (Anther D_RMS_ – Forceps D_RMS_) between forceps and anther for each anther type and species across samples.

Species	Focal anther				Non-focal anther	
	Range	Mean ± s.e.	N	Range	Mean ± s.e.	N
*Cyclamen persicum*	− 33.393 to 16.491	1.343 ± 2.8	19	− 37.727 to 32.433	− 2.476 ± 3.597	19
*Exacum affine*	− 45.133 to 29.824	− 1.312 ± 2.516	24	− 13.069 to 154.366	36.556 ± 10.989	24
*Solanum dulcamara*	− 33.586 to 15.332	− 1.676 ± 2.699	17	− 32.923 to 15.337	− 2.017 ± 2.596	17
*Solanum houstonii*	− 15.2 to 59.12	13.806 ± 4.079	18	7.286 to 174.053	55.098 ± 9.949	18

Values for the vector are calculated from the x- and y-axes (see “Materials and methods” section for details).

*N* number of flowers.

## Discussion

Our study suggests that the arrangement of poricidal anthers affects the transmission of vibrations between anthers. We found that vibrations are transmitted similarly, in both frequency and amplitude, across focal and non-focal anthers in species with stamens partially or totally fused to form a cone (*S. dulcamara* and *C. persicum*). In contrast, species in which individual stamens can move freely (*E. affine* and *S. houstonii*) showed identical frequency but higher vibration amplitudes at the tip of non-focal anthers compared to the focal anthers where vibrations were applied. Overall, the highest displacements occurred in the long anthers of the heterantherous *S. houstonii*. Our work shows that floral architecture, including the functional fusion of stamens into an anther cone, affects the transmission of vibrations applied to a subset of anthers. Because buzz-pollinating bees often grasp with their mandibles and contact with their thorax or abdomen only one or few anthers during buzz pollination, and because pollen release is a function of vibration amplitude, our results suggest that stamen architecture is an important determinant of the functional consequences of the applied vibrations.

The dominant frequency of artificial vibrations did not change as they were transmitted through flowers, regardless of flower type or vibration characteristics. This result aligns with Brito et al.^43^ who also found that artificial vibration dominant frequency is conserved throughout the heterantherous flowers of *S. rostratum* Dunal, both at anther tips and petals. Although some plant substrates such as stems can act as frequency filters^4^, differentially attenuating vibrations components depending on their frequency, frequency is not altered over the short distances involved in vibration transmission during buzz-pollination interactions^20^. Although the natural frequency of anthers is affected by their morphology and organisation within the flower^31^, the frequency of vibrations has limited effects on pollen release in buzz-pollinated flowers, suggesting that resonance plays a minor role within the range of frequencies produced by most bees (100 to 400 Hz)^17,20,31^.

In contrast, we found that the amplitudes of artificial vibrations were differentially altered as they travelled through the two types of buzz-pollinated flowers. In the flowers with more loosely arranged androecia, *E. affine* and *S. houstonii*, vibrations at the tip of the non-focal anther had generally higher displacement amplitude, i.e. moved further, than those observed in the tip of anthers being vibrated. This effect was strongest in the heterantherous *S. houstonii* where, in some cases, displacement was doubled between input and the longer, non-focal pollinating anther. In *S. rostratum*, velocity amplitude from the vibration source to the anther tips of both feeding and pollinating anthers increases up to four-fold when vibrations were applied at the base of the flower^43^. Stamens can be thought of as a complex cantilever beam, a structure with one fixed end and one free end^50^. Vibration displacement amplitude at the tip of the stamen should be partly a function of the stamen’s length, second moment of area, Young’s modulus of elasticity, and mass^51^. Based on cantilever theory, we expect longer stamens to show generally higher displacements at the tip than shorter stamens. Stamen length differences may help explain the difference in vibration amplitude between the short anthers of *E. affine* and the long pollinating anthers of *S. houstonii*. However, stamen material properties, morphology and architecture are likely to affect important parameters determining their vibrational properties, including their second moment of area and Young’s modulus (stiffness)^51^, and predictions based on length alone might not capture the behaviour of real stamens^51^. Previous empirical work shows that amplitude has a significant, positive effect on pollen release^10,16,18^, with increased anther acceleration causing pollen grains to gain in energy and escape through the pores at a higher rate^52^. Clearly more work in this area is needed, including both empirical and modelling studies of the vibrational properties of stamens incorporating the complexity of the forms and material properties of stamens.

Unlike the heterogenous vibration amplitude observed between focal and non-focal anthers of species with loosely held stamens, species in which anthers are held together forming tight, connivent, anther cones (*C. persicum* and *S. dulcamara*) showed vibrations of the same, uniform amplitude between focal and non-focal anthers. The functionally cohesive androecium in these species appears to homogeneously transmit vibrations across the anther cone. The uniformity of the amplitude and frequency of vibrations across all anthers of species with fully or partly fused (connate or connivent) anther cones might have implications for patterns of pollen release during buzz pollination. Species with connivent anther cones may show a more uniform rate of pollen release from each anther when vibrated, compared to the more heterogenous range of vibrations experienced by individual anthers of species in which anthers move more freely. Anther cones have evolved in a variety of taxa with buzz-pollinated flowers including species in the families Ericaceae, Gesneriaceae, Melastomataceae, Primulaceae, Rubiaceae, and Solanaceae^10,24,36,53^, providing excellent opportunities to compare the functional significance of convergent floral morphologies. The same putative uniform pollen release may also occur when non-poricidal anthers are enclosed in a corolla and flowers are buzz-pollinated, as seen in some *Pedicularis* species^11,54^. Our study did not investigate pollen release patterns in different types of flowers and further work quantifying vibratory pollen release in flowers with disparate morphologies across taxonomic groups could help establish the functional consequences, if any, of different androecium architectures.

We suggest that the differences in vibration transmission we see in this study are largely due to differences in stamen architecture in our chosen flower types. However, other morphological differences between the four species are also likely to be important in determining vibration transmission. Studies on other types of insect vibrations have shown that flexible plant stems attenuate vibrations more than stiff stems, as do thick leaves compared with thin leaves^4,5^. In buzz-pollinated flowers, traits affecting vibration properties might include anther curvature (e.g. *S. houstonii*), stamen stiffness and length^31^. Similarly, the size of the anther locules (where the pollen is located before release) and thickness of the anther walls may affect vibration transmission. Few studies have examined the effect of specific morphological traits on vibration transmission in buzz-pollinated flowers, but closely-related species of *Solanum* with similar morphologies can differ in their vibration transmission properties^35^. Moreover, partial removal of stamen structures, such as the connective appendages in *Huberia bradeana* (Melastomataceae), can affect the relative amplitude of vibrations^55^. Although the species studied here differed in anther architecture and the transmission of vibrations through the androecium, all of them have stamens positioned relatively closely together, more or less forming a cone. Other buzz-pollinated species can have stamens more widely separated and not forming a cone, such as those found in several species of Melastomataceae^56^. Given the wide range of morphologies of buzz pollinated flowers^6,20,57^, we expect that a greater difference in vibration transmission could be found in species with more disparate morphologies than those studied here.

We hypothesise that differences in the transmission of vibrations observed here among species with “tight cone” vs. “loose cone” stamen architectures have functional implications for the interaction with buzz pollinators and for patterns of pollen release. If stamen architecture type affects vibration transmission and pollen release patterns, bee pollinators may display different behavioural strategies to buzz these different flowers and maximize pollen removal, for example, by changing the manipulation of anthers during visitation. For instance, we predict that bees on flowers with loose anther arrangements might learn to simultaneously manipulate and buzz multiple anthers if this resulted in more efficient vibration transmission and thus a higher rate of pollen collection^58^. In contrast, bees visiting flowers with anthers that form a tight cone may be able to extract pollen from all anthers regardless of which and how many anthers are manipulated.

From the plant perspective, the uniform vs. heterogenous transmission of vibrations from focal to non-focal anthers in species with cone vs. loose stamens could also have fitness consequences. On the one hand, efficient transmission of vibrations from focal to non-focal anthers could increase pollen deposited on pollinators during single visits, potentially increasing pollen export to other flowers. On the other hand, although not observed here, vibration damping from focal to non-focal anthers could limit the amount of pollen removed from the flower during single visits and increase the release of pollen over multiple visits (pollen dispensing)^18^. The fitness consequences of these patterns of pollen release may also depend on the relative size of the interacting flower and pollinator. Bees that are small relative to the flower are often unable to buzz all anthers at once. To the extent that the visitor is too small to be a legitimate pollinator^59^, reducing pollen release in non-focal anthers (for example by limiting the vibration amplitude of non-focal anthers) may limit pollen loss during visitation by floral larcenists. Both the different stamen arrangements in cone vs. loose stamens and the associated changes in floral handling by visiting pollinators might also influence the precision of pollen placement on bees’ bodies^24^, the placement of pollen of “safe sites”, and thus the efficiency of pollen transfer to stigmas^28,60^. Further studies of how vibrations are applied to flowers with different stamen architectures and their effect on pollen release, including their placement on pollinators’ bodies, in both laboratory and field settings, will help ascertain the functional consequences of the enormous morphological diversity observed in buzz-pollinated flowers.

## Supplementary Information

Supplementary Information.

## Data Availability

The dataset generated and analysed in this study is available in DataStorre: http://hdl.handle.net/11667/174.
