# Supplementary material for: Transmission of bee-like vibrations in buzz-pollinated plants with different stamen architectures
Source: Sci Rep. 2021 Jun 29;11:13541. doi: 10.1038/s41598-021-93029-7 (PMC8241880; doi:10.1038/s41598-021-93029-7)

# Transmission of bee-like vibrations in four species of buzz-pollinated plants with different stamen architectures

Lucy Nevard^1,^ *

Avery L. Russell^2^

Karl Foord^3^

Mario Vallejo-Marín^1^

^1^ Biological & Environmental Sciences. University of Stirling, Stirling, UK FK9 4LA

^2^ Department of Biology. Missouri State University, Springfield, MO 65897

^3^ Minnesota Extension. University of Minnesota, St Paul, MN 55108

* Author for correspondence. Email: lucy.nevard1@stir.ac.uk

**Supplementary table 1.** Linear model for the x axis fitted with D_RMS_ as response, and forceps D_RMS_, anther type and species as fixed effects. **P-*value of fixed effect in linear model. ***P-*value calculated using Type III sums of squares. Sample size is 150.

| **X axis** | **Estimate** | | | **Std. error** | ***P*-value*** | ***P*-value**** |
| --- | --- | --- | --- | --- | --- | --- |
| **Displacement amplitude D_RMS_ (µm)** | |  |  | |  |  |
| Forceps D_RMS_ | 0.946 | | 0.056 | | **<0.001** | **<0.001** |
| Anther (Non-focal) | -7.696 | | 24.43 | | 0.078 | 0.753 |
| Species |  | |  | |  | 0.846 |
| (*Exacum affine*) | 9.708 | | 20.738 | | 0.640 |  |
| (*Solanum dulcamara*) | 7.982 | | 22.914 | | 0.728 |  |
| (*Solanum houstonii*) | 18.722 | | 21.106 | | 0.887 |  |
| Forceps D_RMS_: Anther (non-focal) | -0.037 | | 0.082 | | 0.652 | 0.652 |
| Anther (non-focal): Species |  | |  | |  | **<0.005** |
| Anther: (*Exacum affine*) | 29.011 | | 29.623 | | 0.329 |  |
| Anther: (*Solanum dulcamara*) | 14.929 | | 32.161 | | 0.643 |  |
| Anther: (*Solanum houstonii*) | 108.014 | | 30.903 | | **<0.001** |  |

| **Y axis** | **Estimate** | | | **Std. error** | ***P*-value*** | ***P*-value**** |
| --- | --- | --- | --- | --- | --- | --- |
| **Displacement amplitude D_RMS_ (µm)** | |  |  | |  |  |
| Forceps D_RMS_ | 1.00849 | | 0.03535 | | **<0.001** | **<0.001** |
| Anther (Non-focal) | -23.404 | | 16.187 | | 0.15 | 0.15 |
| Species |  | |  | |  | **<0.005** |
| (*Exacum affine*) | 0.207 | | 13.919 | | 0.988 |  |
| (*Solanum dulcamara*) | -11.809 | | 15.43 | | 0.445 |  |
| (*Solanum houstonii*) | 35.077 | | 14.159 | | **<0.05** |  |
| Forceps D_RMS_: Anther (non-focal) | 0.15 | | 0.0514 | | 0.004 | 0.004 |
| Anther (non-focal): Species |  | |  | |  | **<0.005** |
| Anther: (*Exacum affine*) | 100.6 | | 19.893 | | **<0.001** |  |
| Anther: (*Solanum dulcamara*) | 3.928 | | 21.628 | | 0.856 |  |
| Anther: (*Solanum houstonii*) | 11.85 | | 20.78 | | 0.569 |  |

**Supplementary table 2.** Linear model for the y axis fitted with DRMS as response, and forceps DRMS, anther type and species as fixed effects. *P-value of fixed effect in linear model. **P-value calculated using Type III sums of squares. Sample size is 150.


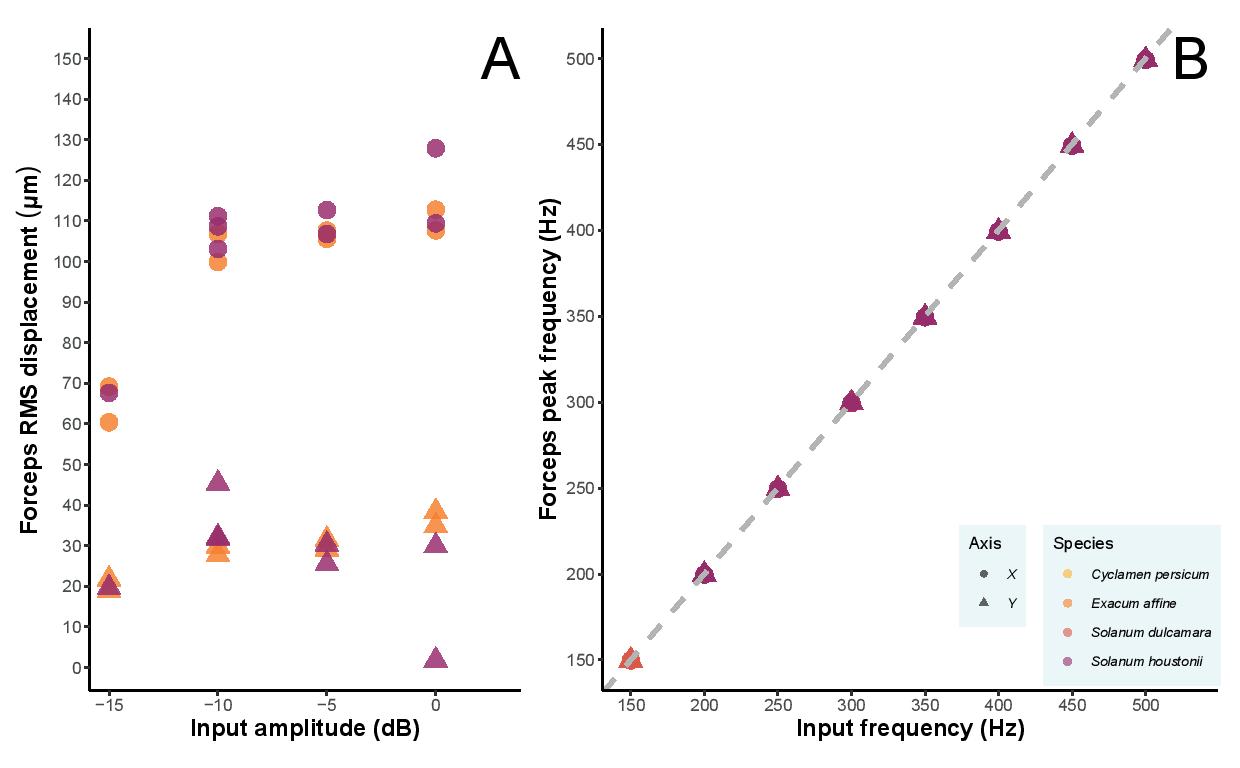
**Supplementary figure 1.** Measured RMS displacement (µm) of forceps against input amplitude (dB) (A) and measured peak frequency (Hz) of forceps against input frequency (Hz) (B). Grey dashed line indicates a linear relationship with slope=1.


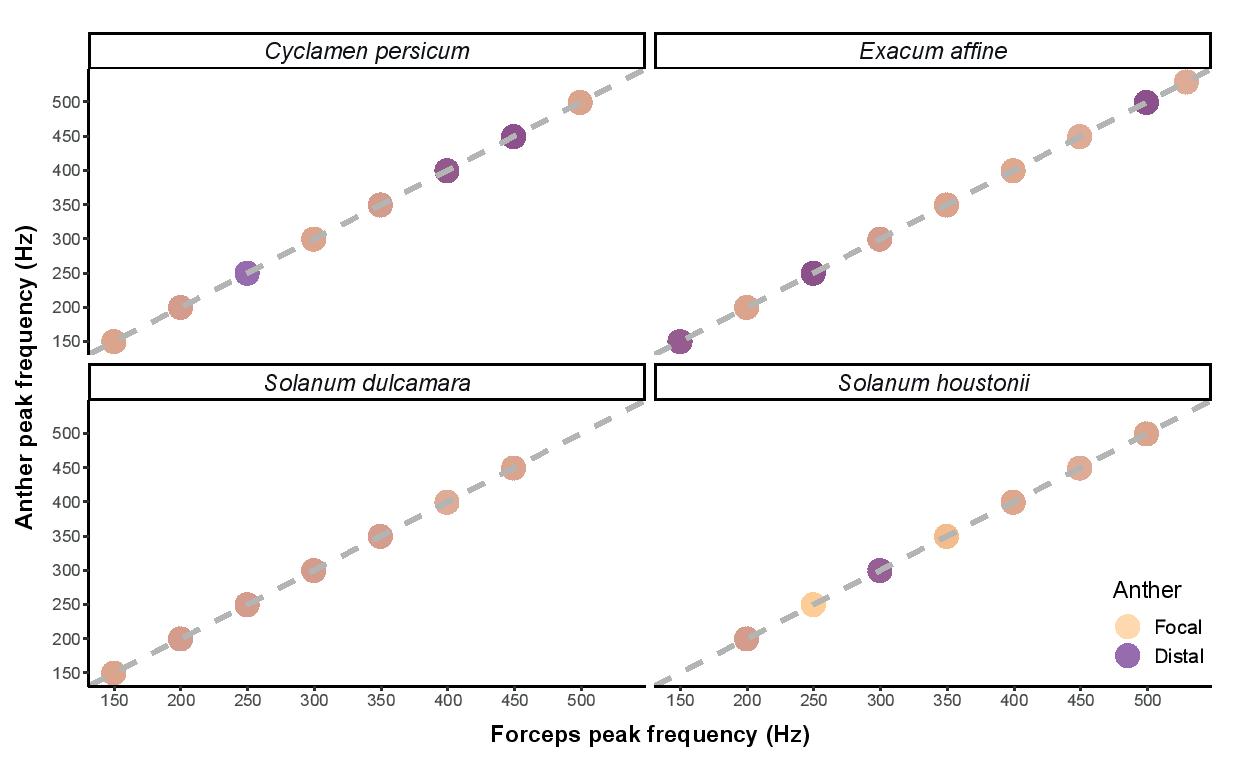


**Supplementary figure 2.** Measured peak frequency (Hz) against forceps frequency (Hz) for focal and distal anther of four plant species. Grey dashed line indicates a linear relationship with slope=1


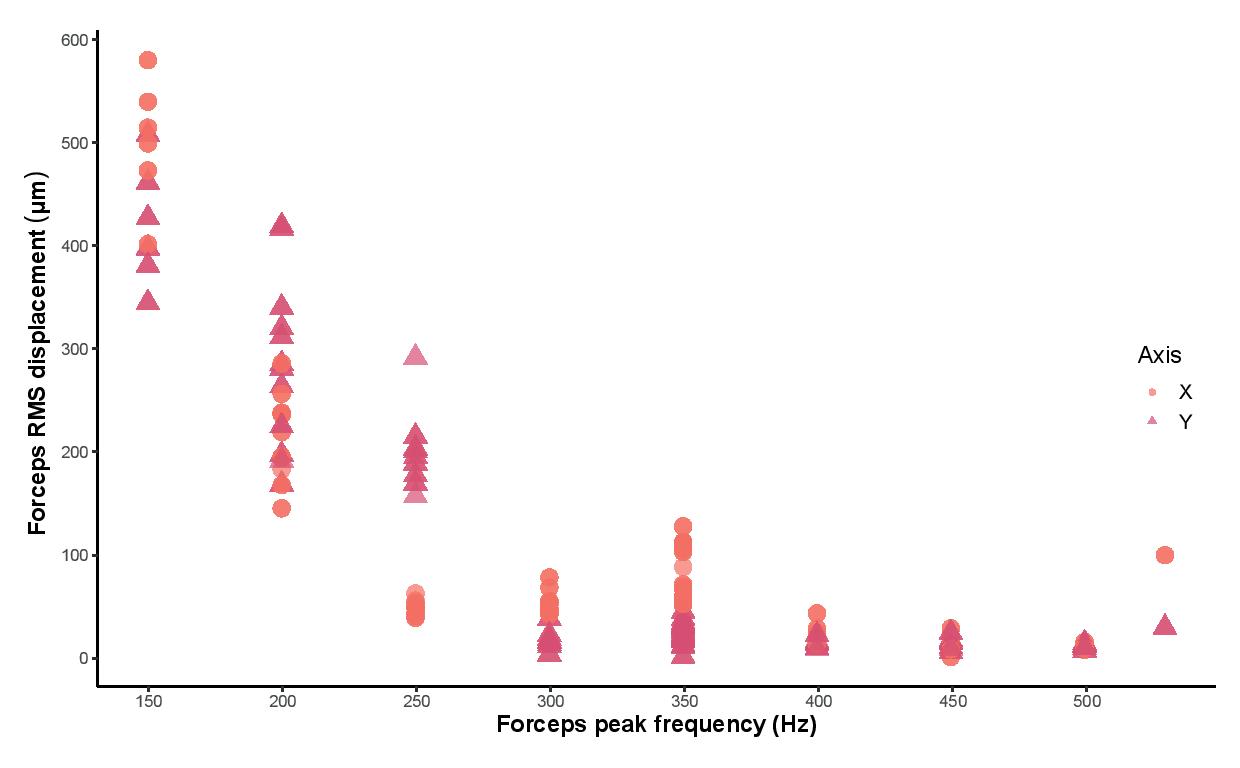


**Supplementary figure 3.** Forceps peak frequency (Hz) v forceps RMS displacement (µm) for both axes.

**Supplementary figure 4.** Anther peak frequency v anther RMS displacement (µm) for both axes.


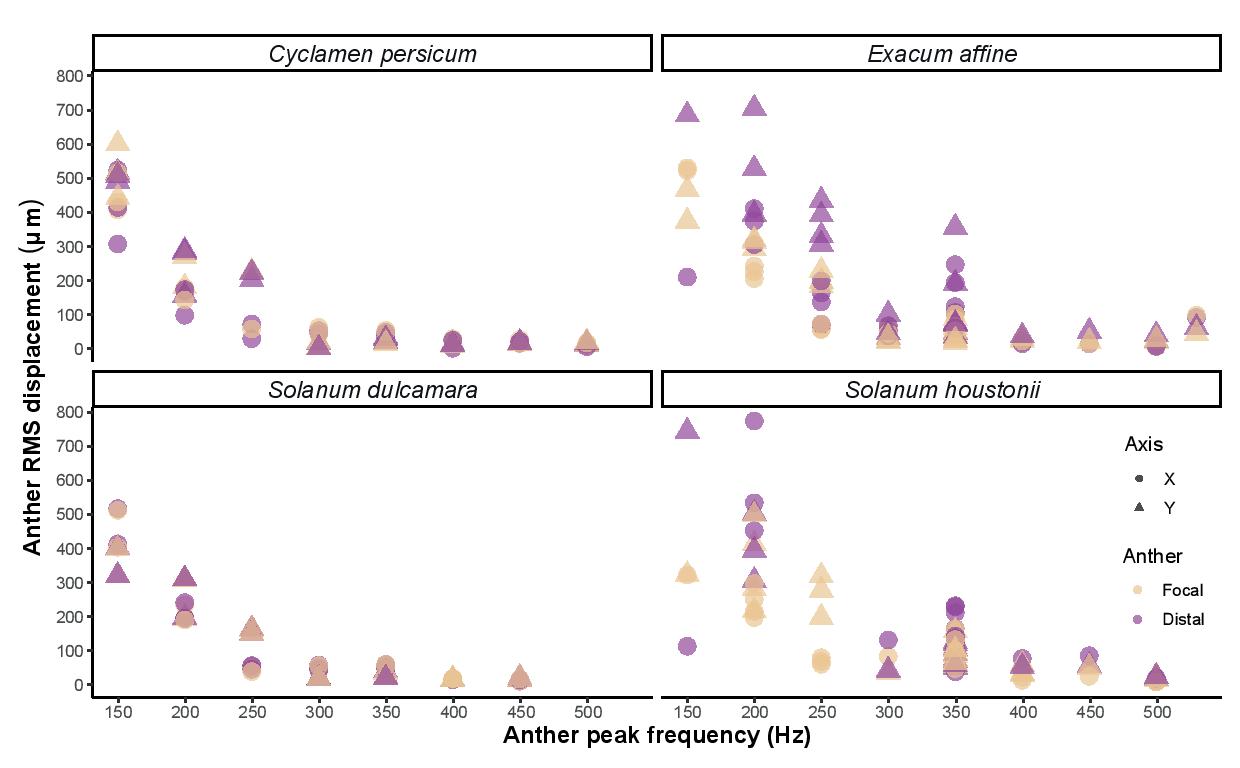


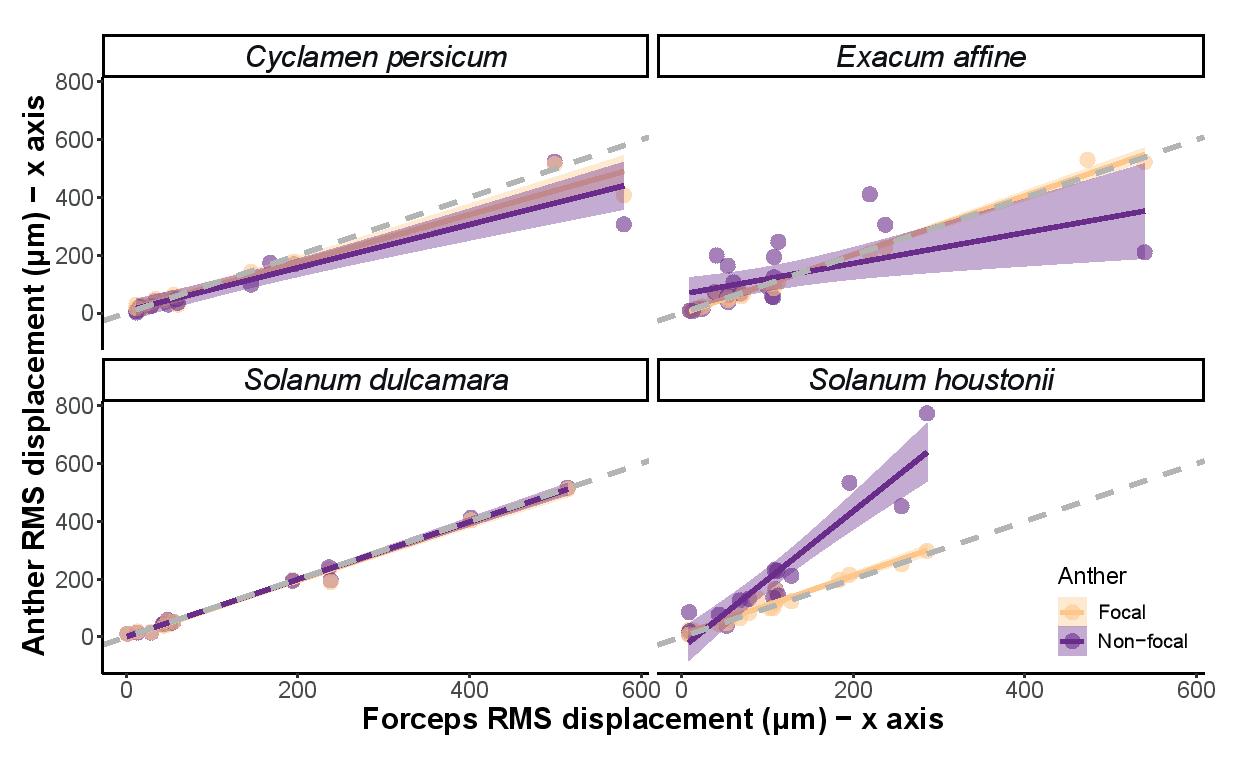
**Supplementary figure 5.** Linear model estimates and data points for measured x-axis RMS displacement (µm) of focal and distal anther against forceps RMS displacement (µm) in four plant species. Grey dashed line indicates a linear relationship with slope=1.

**Supplementary figure 6.** Linear model estimates and data points for measured y-axis RMS displacement (µm) of focal and distal anther against forceps RMS displacement (µm) in four plant species. Grey dashed line indicates a linear relationship with slope=1.
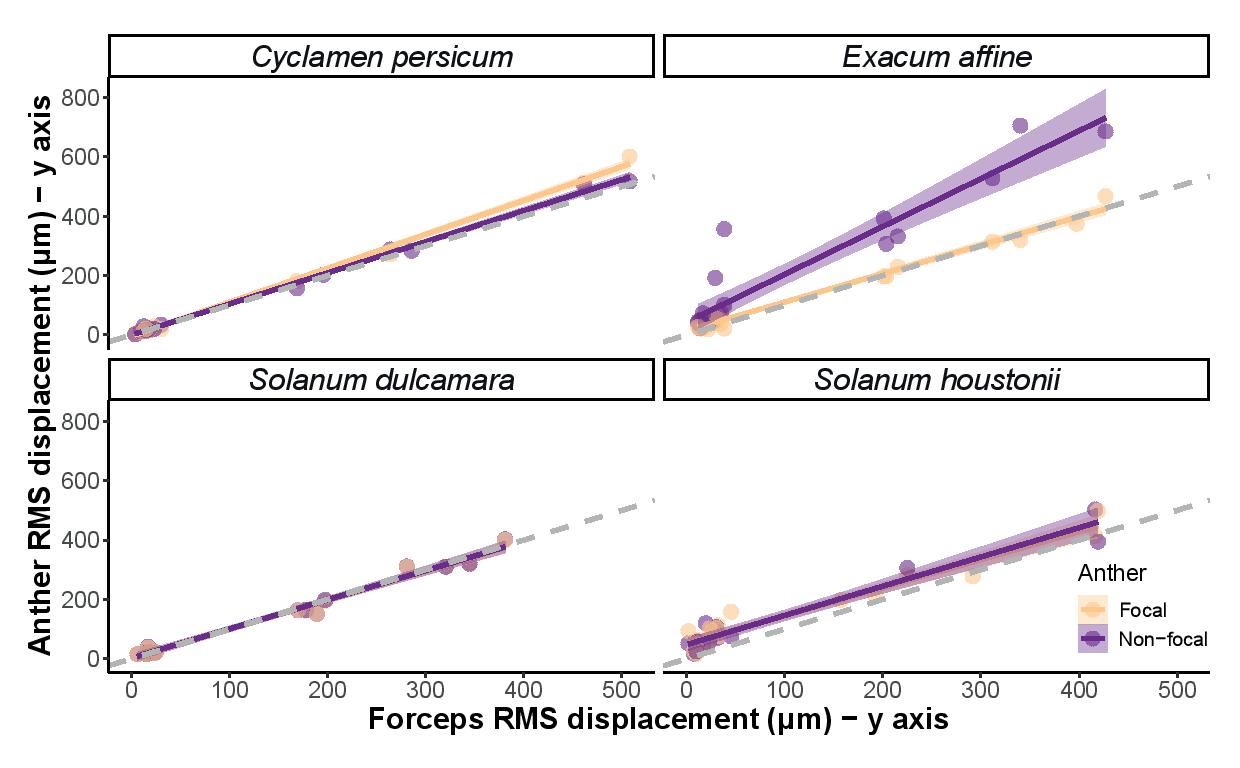

Supplement: Supplementary file 1 — Supplementary Information. [file 41598_2021_93029_MOESM1_ESM.docx]
